# On the Re-production of the Crystalline Lens

**Published:** 1845-01

**Authors:** 


*On the Re-production of the Crystalline Lens.*—The following is a summary of the conclusions to which M. Textor, in his recent dissertation, Ueber die Wiederzeugung der Krystallinse, has arrived.

The extraction or depression of the crystalline lens is followed by the re-production of a new one that is more or less regular in its configuration, or at least by the formation of a certain portion of the crystalline mass.—This re-production is the work of the investing capsule, provided this remains uninjured.—When the capsule is removed, the lens is not re-produced.—The capsule adheres to the new crystalline ring (*bourrelet*), but not so very closely that they cannot be separated.—The newly-formed lens possesses the same transparency as the old one.—The re-production of the lens requires a certain length of time, which is however very variable in different circumstances.—The new lens gradually acquires a greater degree of solidity and thickness.—Its form depends much on the extent of lesion of the capsule.—The capsule has, in all cases of re-production of the lens, been observed to retain its transparency.—*Med. Chir. Rev.*

